# Systematic Review of Cysteine-Sparing *NOTCH3* Missense Mutations in Patients with Clinical Suspicion of CADASIL

**DOI:** 10.3390/ijms18091964

**Published:** 2017-09-13

**Authors:** Elena Muiño, Cristina Gallego-Fabrega, Natalia Cullell, Caty Carrera, Nuria Torres, Jurek Krupinski, Jaume Roquer, Joan Montaner, Israel Fernández-Cadenas

**Affiliations:** 1 Stroke Pharmacogenomics and Genetics, Fundació Docència i Recerca Mútua Terrassa, Hospital Mútua de Terrassa, 08221 Terrassa, Spain; elena.muinho@gmail.com (E.M.); cristina.gallego.fabrega@gmail.com (C.G.-F.); natalia.cullell@gmail.com (N.C.); n.torres.ag@gmail.com (N.T.); 2 Neurovascular Research Laboratory, Vall d’Hebron Institute of Research, Hospital Vall d’Hebron, 08035 Barcelona, Spain;catycarrerav@gmail.com (C.C.); joan.montaner@vhir.org (J.M.); 3 Neurology Service, Hospital Mútua de Terrassa, 08221 Terrassa, Spain; jkrupinski@mutuaterrassa.es; 4 Neurology Service, Institut Hospital del Mar d’investigacions Mèdiques, IMIM-Hospital del Mar, 08003 Barcelona, Spain; JRoquer@parcdesalutmar.cat

**Keywords:** CADASIL, cysteine, *NOTCH3*, mutation, temporal pole

## Abstract

CADASIL (cerebral autosomal dominant arteriopathy with subcortical infarcts and leukoencephalopathy) is caused by mutations in the *NOTCH3* gene, affecting the number of cysteines in the extracellular domain of the receptor, causing protein misfolding and receptor aggregation. The pathogenic role of cysteine-sparing *NOTCH3* missense mutations in patients with typical clinical CADASIL syndrome is unknown. The aim of this article is to describe these mutations to clarify if any could be potentially pathogenic. Articles on cysteine-sparing *NOTCH3* missense mutations in patients with clinical suspicion of CADASIL were reviewed. Mutations were considered potentially pathogenic if patients had: (a) typical clinical CADASIL syndrome; (b) diffuse white matter hyperintensities; (c) the 33 *NOTCH3* exons analyzed; (d) mutations that were not polymorphisms; and (e) Granular osmiophilic material (GOM) deposits in the skin biopsy. Twenty-five different mutations were listed. Four fulfill the above criteria: p.R61W; p.R75P; p.D80G; and p.R213K. Patients carrying these mutations had typical clinical CADASIL syndrome and diffuse white matter hyperintensities, mostly without anterior temporal pole involvement. Cysteine-sparing *NOTCH3* missense mutations are associated with typical clinical CADASIL syndrome and typical magnetic resonance imaging (MRI) findings, although with less involvement of the anterior temporal lobe. Hence, these mutations should be further studied to confirm their pathological role in CADASIL.

## 1. Introduction

CADASIL (cerebral autosomal dominant arteriopathy with subcortical infarcts and leukoencephalopathy) is an inherited systemic arterial vessel disease caused by mutations in the *NOTCH3* gene, located on the 19p13 chromosome [1,2], which encodes a transmembrane receptor that is mainly expressed in the smooth muscle cells of blood vessels and pericytes [3].

This receptor has three domains: (1) a large extracellular domain (ECD) with 34 epidermal growth factor (EGF)-like repeats encoded by exons 2–24, where *NOTCH3* mutations are typically located; (2) a transmembrane domain; and (3) an intracellular domain (ICD). Each EGF-like repeat contains six cysteines that form three disulfide bonds, which contribute significantly to the protein’s tertiary structure.

Pathogenic mutations in CADASIL are dominant; the presence of only one mutation in one of both alleles is the cause of the pathology. The pathogenic mutations are associated with changes in the number of cysteines, these changes cause an odd number of cysteines, leading to a misfolding of the receptor, enhanced formation of oligomers and ECD aggregation, facilitated by crosslinking of sulfhydryl groups [4,5,6]. This ECD aggregation is considered to be the main pathogenic mechanism of the disease. In terms of NOTCH3 activity, classic mutations have been described with normal or decreased NOTCH3 activity. Nevertheless, its role in the pathogenic mechanism is controversial, and several authors have shown that altered NOTCH3 function is not the primary determinant of the disease [7,8,9].

The main symptoms of CADASIL are migraine, psychiatric disorders, recurrent small subcortical infarctions, and dementia [10]. The average age at stroke onset is approximately 46 years, although recent studies have described an older onset, at 52 years for males and 57 for females [11]; for dementia, the average age at onset is 55 [12].

Magnetic resonance imaging (MRI) and histopathology show small subcortical infarctions and confluent white matter hyperintensities (WMH), mostly in periventricular locations and with additional involvement of the anterior temporal pole and the external capsule [13].

It has been described elsewhere that WMH in the temporal pole have 89–95% sensitivity and 80–86% specificity for CADASIL diagnosis in Caucasian patients [14], and up to 95% of these patients have this alteration [15]. Nevertheless, in Asian populations the prevalence of temporal pole involvement is only between 44.8–71% [16].

Additionally, it is typical to find granular osmiophilic material (GOM), consisting of parts of the extracellular domain of NOTCH3 [17], in the extracellular space and located close to the cell surface in smooth-muscle cells [18]. The presence of GOM has 45–96% sensitivity and 100% specificity for CADASIL diagnosis [15,19], but the pathological role of GOM is still debated.

Recent studies have found that mutations that do not affect the number of cysteines (unlike the typical mutations) seem to be associated with clinical CADASIL syndrome. However, the pathogenic role of these mutations is uncertain.

Therefore, this is a systematic review of those studies in which patients with a clinical suspicion of CADASIL and cysteine-sparing *NOTCH3* missense mutations have been described. The aims of this article are to: (1) find data in the literature and in databases relating to cysteine-sparing *NOTCH3* missense mutations observed in patients with typical clinical CADASIL syndrome; (2) describe the epidemiological characteristics of these patients; (3) determine whether these mutations could be considered potentially pathogenic.

There is not a consensus to determine whether a mutation can be considered pathogenic. Depending on the disease the presence of a missense mutation that has not been described previously in healthy subjects and with bioinformatic data supporting the pathogenicity is enough to consider a mutation the cause of the disease [20]. However this is not the case for other diseases and more analysis should be performed. For instance in neurodegenerative disorders there are studies that have analyzed the conservation of the residue during the evolution or have analyzed the presence of described mutations involving the same Wild-type amino acid [21,22].

In the case of CADASIL, the presence of GOM is very important because this finding indicates that the patient has CADASIL. The other critical point is to analyze all the exons of the *NOTCH3* gene to find the mutation that causes the pathology. In our systematic review we have considered these two analyzes among others to identify a pathogenic mutation.

## 2. Results

Of the 244 studies reviewed, 16 fulfilled the inclusion/exclusion criteria and 34 index cases were described, with 25 different mutations. Twelve out of the 18 patients were female (67%) and six were male (33%); 17 of the total number of patients were European (50%), 14 were Asian (41%), two patients were from Oceania and one was North-American (Table 1).

This table shows the main genetic features of the 34 index patients: mutation, amino acid replaced, amino acid substituent, exon where mutation is located, exons sequenced, minor allele frequency (MAF) according to Exome Aggregation Consortium (ExAC), MAF according to 1000 Genomes Project, author and reference.

### 2.1. Mutation Features

Twenty-five different cysteine-sparing *NOTCH3* missense mutations were identified from the 34 patients described in the 18 papers analyzed (Table 1). A complete evaluation of the *NOTCH3* exons was carried out in 29% (8 out of 28) of the patients; therefore, in the other cases it was not possible to rule out mutations involving cysteine residues. The mutations with the 33 exons analyzed were: p.R61W [23]; p.R75P [25]; p.D80G [27]; p.G149V [29]; p.R213K [33]; p.L1515P [37]; and p.V1762M [38] (Table 1).

Amino acid changes involving arginine were present in 44% of the patients, while those involving proline were present in 26%. Only two mutations were found in exons that do not encode for the ECD: p.L1515P [37] and p.V1762M [38], which encoded for the ICD.

The search for all mutations in Exome Aggregation Consortium (ExAC) and the 1000 Genomes Project showed that only p.H1235L [28], p.H170R [30,31], p.S497L [34], p.A1020P [36], and p.H1133Q [34] were polymorphisms or subpolymorphic variants, as their MAF was >0.1%.

### 2.2. Clinical Features

Of the 34 probands described in the 18 articles, 33% presented cardiovascular risk factors such as smoking, hypertension, and/or dyslipidemia. Clinical symptoms included migraine (93%), stroke (100%), seizures (60%), psychiatric disturbances (100%), pseudobulbar palsy (80%), and dementia (94%), and gait disturbance was specified in four cases. A family history of stroke and/or dementia was observed in 96% of the probands (Table 2).

The neuroimaging data revealed that all probands had severe WMH, although 91% did not have anterior temporal pole involvement.

GOM deposits were found in six out of nine probands (67%). In two of these cases, they were also found in the proband’s siblings. Specifically, mutations with GOM were: p.R61W [23] in the symptomatic proband’s siblings, who had the same mutation; p.R75P [24,25,26]; p.D80G [30] in the symptomatic proband’s siblings, who had the same mutation; p.R213K [33], p.A1020P [36] (in one of the two probands); and p.T1098S [26]. However, three out of nine probands had cysteine-sparing *NOTCH3* missense mutations not associated with GOM: p.A1020P [33] (in one of two probands), p.L1515P [34] and p.V1762M [35]; the latter two were mutations located in the ICD.

A summary of the main characteristics of cysteine-sparing *NOTCH3* missense mutations is given in Table 3.

The table not only summarizes the main characteristics of cysteine-sparing *NOTCH3* missense mutations, but also shows the five criteria required for mutations to be considered potentially pathogenic: type of mutation, typical clinical CADASIL syndrome, diffuse WMH, whole exon analysis, mutations that were not polymorphisms and GOM deposition, and the author.

Therefore, we considered that p.R61W [23], p.R75P [25], p.D80G [27], andp.R213K [33] could be potentially pathogenic mutations, because they were associated with typical clinical CADASIL syndrome and extensive WMH in MRI, the 33 *NOTCH3* exons were analyzed and no other potential pathogenic mutations were found; they had an MAF < 0.1% (ExAC, 1000 Genomes Project) (confirming that they are not common polymorphisms) and GOM deposits were observed in the skin biopsy. (The criteria for considering mutations as potentially pathogenic are explained in the Material and Methods section).

In relation to co-segregation of these potentially pathogenic mutations, Mizuno et al. [25] studied the relatives of probands with the p.R75P mutation. Those with clinical CADASIL and typical MRI findings had the same p.R75P mutation, and the asymptomatic relative did not carry the mutation. For p.D80G, two sisters and one brother with clinical CADASIL typical syndrome and extensive WMH shared the same mutation, and the other asymptomatic brother did not share it. In addition, the symptomatic brother had GOM in his skin biopsy, unlike the asymptomatic one. Segregation analysis was not performed for p.R61W or p.R213K.

Of the ten probands that carried one of these four mutations, 80% were Asian, 60% had the p.R75P mutation and just one of the probands (p.D80G) had temporal pole involvement.

Polyphen-2 analysis indicated that p.R61W, p.D80G, and p.R75P could be possible damaging mutations (Table 4).

## 3. Discussion

In this systematic review, we conducted a thorough search for cysteine-sparing *NOTCH3* missense mutations in patients with typical clinical CADASIL syndrome in order to determine whether these mutations could be considered pathogenic and to describe the patients that carry this type of mutation.

We excluded nonsense and insertion/deletion mutations because they lead to a numerical cysteine alteration, as Rutten et al. described in a previous article [8].

The literature and gene databases describe 34 patients with 25 different cysteine-sparing *NOTCH3* missense mutations. These 25 mutations included two in exons that encode for the ICD. The patients presented typical clinical CADASIL syndrome with migraine, psychiatric disturbances, early onset of stroke and/or dementia, and diffuse WMH in the MRI.

We believe there are four possible pathogenic cysteine-sparing *NOTCH3* missense mutations: p.R61W [23], p.R75P [25], p.D80G [27], and p.R213K [33]. These mutations were located in EGF-like repeats. We think they are possible pathogenic mutations because patients presented typical clinical CADASIL syndrome and MRI profiles, no additional mutations were found in the gene, they do not represent low-frequency polymorphisms or subpolymorphic variants, and GOM deposits were found in their skin biopsy. A familial co-segregation was observed for p.R61W [23], p.D80G [27] and p.R75P [8,25], but not for p.R213K, because symptomatic relatives were not evaluated. In addition, Polyphen-2 analysis predicted that p.R61W, p.D80G, and p.R75P, but not p.R213K could be possibly damaging mutations. Taking into account the information published in the literature these mutations can be considered possible pathogenic mutations.

Of the 10 probands with these four mutations and CADASIL patients with cysteine mutations, non-significant clinical or demographic differences were observed in migraine, stroke, or dementia. However, WMH in the anterior part of the temporal pole were practically absent in these cysteine-sparing *NOTCH3* missense mutations. As mentioned previously, WMH in the temporal pole has a reported 89–95% sensitivity and 80–86% specificity for CADASIL in Caucasian patients [14] and a prevalence of 44.8–71% [16] in Asian populations. Therefore, considering that 80% of patients in the review were Asians, at least four to five patients with temporal pole involvement should be expected in CADASIL due to cysteine mutations. Therefore, we believe that absent temporal pole involvement could be a characteristic of cysteine-sparing *NOTCH3* missense mutations.

Rutten et al. performed an extensive search of all types of *NOTCH3* mutations in order to provide information on their interpretation [8]. Regarding the cysteine-sparing *NOTCH3* missense mutations, they studied 10 articles with 11 different mutations corresponding to p.R61W, p.R75P, p.Q151E, p.H170R, p.A202V, p.R213K, p.V237M, p.T577A, p.S978R, p.A1020P, and p.Y1098S. They concluded that these mutations were not associated with CADASIL, but p.R75P fulfilled all their criteria: (1) a complete evaluation of the *NOTCH3* gene to rule out a typical cysteine-altering mutation in another exon; (2) the mutations were not polymorphisms; (3) a familial co-segregation was proven; and (4) the clinical diagnosis was confirmed through the observation of GOM deposits in the skin biopsy.

This systematic review updates the interesting work of Rutten et al. [8], taking into consideration three of the four criteria that they applied, excluding the lack of familiar segregation criterion [8]. We were able to add new information and conclusions stemming from the publication of new articles with a total of 25 different mutations, in contrast to the 11 found previously by Rutten et al. Of the four mutations considered potentially pathogenic in our systematic review, p.R75P was already mentioned by Rutten et al. in their reference to Mizuno et al. In this review, we added information from two new articles with a total of four new patients. Regarding p.R213K, Rutten et al. did not consider this mutation as potentially pathogenic because familial co-segregation was not proven. However, we did not define familial co-segregation as an exclusion criterion because it is not always possible to analyze relatives or there may be no live relatives.

The genetic analysis technique was not specified for p.R61W, so Rutten et al. suggested the possibility of using a technique that would not detect other mutations.

Finally, p.D80G was not studied previously by Rutten et al., but applying their criteria and ours, it is a pathogenic mutation.

In several cases, the conclusions of these articles are supported by bioinformatics and functional studies that have revealed the possible pathogenic effect of the mutations [37,39]. Mizuno et al. suggested that changes in NOTCH3 structure could be related to the replacement of an amino acid by proline (without cysteine involvement), because three-dimensional structure studies have shown that it helps to stabilize the β-sheet [25]. This may lead to a conformational change in the protein. Another study observed that several of these atypical mutations were associated with structural changes in the NOTCH3 receptor [39], similar to the mechanism observed for typical mutations involving cysteines. Thus, in addition to changes in the number of cysteines, mutations involving other amino acids could also lead to misfolding of the receptor. In the same way, in vitro studies with p.R75P, p.D80G, and delta88–91 have shown significantly enhanced aggregation similar to that of cysteine mutations [27].

With regard to limitations, we should consider the low number of cases reported so far, as well as the high number of Asian reports that could skew the frequencies of different features in this population. In order to avoid this last limitation, we compared some important characteristics (such as the temporal pole affectation) to their frequency in the Asian population.

Another limitation is that the analyses of p.R213K [33] to rule out other mutations in the *NOTCH3* gene were performed using single-strand conformational polymorphism (SSCP) analysis, which does not detect other mutations, unlike the standard and reliable Sanger sequencing. In the case ofp.R61W, the genetic analysis technique was not specified.

Furthermore, we did not consider a small deletion (delta88–91) that does not directly involve a cysteine residue in the ECD of NOTCH3 [39]. This would have enriched our review, but could have compromised the reliability of our conclusions. This deletion was reported in an Italian family with typical clinical CADASIL syndrome and diffuse WMH, without anterior temporal pole involvement. In addition, the proband’s skin biopsy showed GOM deposits. This mutation was also observed in the only symptomatic relative. The asymptomatic relatives did not carry any *NOTCH3* mutations.

Another limitation was combining ECD and ICD mutations in our review. The aim here was to review typical clinical CADASIL syndrome with cysteine-sparing *NOTCH3* missense mutations to determine whether they were associated with CADASIL. In any case, ICD mutations were not considered pathogenic because GOM deposits were absent.

## 4. Materials and Methods

An extensive literature search was performed up to November 2016 on PubMed, Google Scholar, EMBASE, LILACS, Trip Database, and the Cochrane Library, as well as in “The Human Gene Mutation Database” (http://www.hgmd.cf.ac.uk/ac/index.php), with the key words: “CADASIL cysteine”, “*NOTCH3* cysteine”, and “*NOTCH3* polymorphism”, in order to collect data about cysteine-sparing *NOTCH3* missense mutations that were associated with typical clinical CADASIL syndrome.

**Figure 1:**
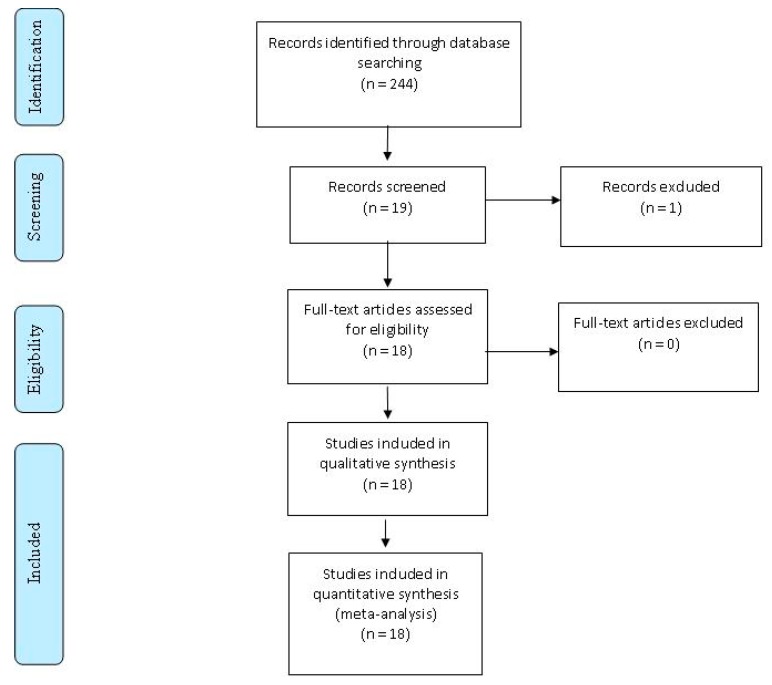
Flow diagram of the literature review. This detailed flow diagram depicts the search and selection processes.

A total of 244 studies were found. Probands had typical clinical CADASIL syndrome and a cysteine-sparing *NOTCH3* missense mutation (Figure 1) in 18 articles. Studies that analyzed other diseases or endophenotypes, such as patients with diffuse WMH instead of a typical clinical CADASIL syndrome, were excluded.

Only those articles that described missense mutations confirming an absence of cysteine involvement were selected. Therefore, nonsense, intronic, insertion, and deletion mutations were excluded because they can lead to cysteine number alterations, through the shortening of the protein (in the case of nonsense mutations) [40,41] and or through changes in the open reading frame (in the case of insertion-deletions) as described previously [8,42].

In addition, we excluded several studies describing mutations that were already known polymorphisms, suggesting a negative pathogenic role, as well as an article by Ueda et al. [16], as it was difficult to identify who was the proband and who was the relative of the patient with the typical clinical CADASIL syndrome.

We collected data on epidemiological, clinical, genetic, and neuroimaging characteristics, such as temporal pole involvement, as well as existence of skin biopsies. We used the IBM SPSS statistics software package, version 22, for the frequencies analysis.

Mutations were considered potentially pathogenic when they fulfilled the following criteria: (1) the patients had typical clinical CADASIL syndrome; (2) the patients had diffuse WMH in MRI; (3) the study analyzed the 33 exons of the *NOTCH3* gene to rule out other pathogenic mutations; (4) the mutation had a MAF < 0.1%, since CADASIL is a low prevalence disease and it is therefore necessary to rule out low-frequency polymorphisms (MAF < 5%) and subpolymorphic variants (frequency 0.1–1.0%) that could represent rare variants and not disease-causing mutations. To establish the MAF, we used the ExAC database, containing genetic information on 60,706 unrelated individuals (http://exac.broadinstitute.org), and the 1000 Genomes Project database, containing the genetic information of 1000 individuals from different ethnicities (http://www.internationalgenome.org/1000-genomes-browsers/); and lastly (5) the patient had GOM deposits in the skin biopsy because this represents a specificity of 100% for CADASIL diagnosis. Finally we analyzed with Polyphen-2 tool the pathogenic prediction of the selected mutations that we considered potentially pathogenic (http://genetics.bwh.harvard.edu/pph2/).

## 5. Conclusions

We believe that the cysteine-sparing *NOTCH3* missense mutations p.R61W, p.R75P, p.D80G, and p.R213K could be potentially pathogenic, and we found familial co-segregation forp.R61W, p.R75P, and p.D80G. Different studies confirmed an altered NOTCH3 structure or proaggregatory properties due to these changes. Thus, cysteine-sparing *NOTCH3* missense mutations are associated with typical clinical CADASIL syndrome and a typical MRI profile, mostly without anterior temporal pole involvement.

Further studies are necessary to clarify the role of these cysteine-sparing *NOTCH3* missense mutations in CADASIL to be able to interpret them properly to reach a correct diagnosis. Likewise, an improved understanding of this kind of mutation would be important to clarify whether other mechanisms, apart from disulfide bond-mediated misfolding due to an odd number of cysteines, play a major role in the development of CADASIL, such as a receptor misfolding produced by other amino acids.

## Figures and Tables

**Table 1:** Mutations found that do not affect cysteine amino acids.

Number	Mutation	Replaced	Substitution	Exon	Sequencing	MAF (ExAC)	MAF (1000 Genomes)	Author
1	p.R61W	Arg	Trp	2	33 exons	0.00007471	-	Brass [23]
2	p.R75P	Arg	Pro	3	Exon: 3, 4, 11, 18; intron	0	0	Kim [24]
3	p.R75P	Arg	Pro	3	Exon: 3, 4, 11, 18; intron	0	0	Kim [24]
4	p.R75P	Arg	Pro	3	Exon: 3, 4, 11, 18; intron	0	0	Kim [24]
5	p.R75P	Arg	Pro	3	33 exons, promotor	0	0	Mizuno [25]
6	p.R75P	Arg	Pro	3	33 exons, promotor	0	0	Mizuno [25]
7	p.R75P	Arg	Pro	3	Exon: 2–24	0	0	Wang [26]
8	p.D80G	Asp	Gly	3	33 exons	0	0	Wollenweber [27]
9	p.R107W	Arg	Trp	3	Exon: 2–23	0.00001750	-	Ungaro [28]
10	p.G149V	Gly	Val	4	33 exons, intron	0	0	Ge [29]
11	p.Q151E	Gln	Glu	4	Exon: 2–23	0.00005703	-	Ungaro [28]
12	p.Q151E	Gln	Glu	4	Exon: 2–6, 8, 11, 14, 18, 19, 22, 23; intron	0.00005703	-	Ampuero [30]
13	p.H170R	His	Arg	4	Exon: 2–6, 8, 11, 14, 18, 19, 22, 23; intron	0.001917	0.0014	Ampuero [30]
14	p.H170R	His	Arg	4	Exon: 2, 3, 4, 11, 18, 19	0.001917	0.0014	Roy [31]
15	p.A198T	Ala	Thr	4	Exon: 2–23	0.00002513	-	Ungaro [28]
16	p.A202V	Ala	Val	4	Exon: 2, 3, 4, 11, 18, 19	0.00001672	-	Roy [31]
17	p.R207H	Arg	His	4	Exon: 2–23	0.00001664	0.0004	Ungaro [28]
18	p.R213K	Arg	Lys	4	-	0	0	Uchino [32]
19	p.R213K	Arg	Lys	4	33 exons	0	0	Santa [33]
20	p.V237M	Val	Met	5	-	0.0002239	0.0008	Uchino [32]
21	p.V252M	Val	Met	5	Exon: 2–23; intron	0.00002493	-	Abramycheva [34]
22	p.E309K	Glu	Lys	6	Exon: 2–23	0	0	Ungaro [28]
23	p.S497L	Ser	Leu	9	Exon: 2–23; intron	0.01234	0.0074	Abramycheva [34]
24	p.T577A	Thr	Ala	11	-	0.00001669	0	Ferreira [35]
25	p.R592S	Arg	Ser	11	Exon: 2–23	0.00006714	-	Ungaro [28]
26	p.V644D	Val	Asp	12	Exon: 2–23	0.0007013	0.0002	Ungaro [28]
27	p.S978R	Ser	Arg	18	-	0.0004606	0.0004	Ferreira [35]
28	p.A1020P	Ala	Pro	19	-	0.07318	0.110	Scheid [36]
29	p.A1020P	Ala	Pro	19	-	0.07318	0.110	Scheid [36]
30	p.T1098S	Thr	Ser	20	Exon: 2–24	0	0	Wang [26]
31	p.H1133Q	His	Gln	21	Exon: 2–23, intron	0.01022	0.0030	Abramycheva [34]
32	p.H1235L	His	Leu	22	Exon: 2–23	0.003990	0.0012	Ungaro [28]
33	p.L1515P	Leu	Pro	25	33 exons, intron	0	0	Fouillade [37]
34	p.V1762M	Val	Met	29	33 exons	0.0002146	-	Bersano [38]

**Table 2:** Clinical and demographic data of the patients with cysteine-sparing *NOTCH3* mutations.

Numb.	Mutation	Origin	Sex	Smoker (*n*=7)	HT (*n* = 15)	DM (*n* = 13)	Dyslip (*n* = 11)	Clin. Onset (Years)	Migraine (*n* = 15)	Stroke (*n* =17)	Seizure (*n* = 5)	Psych. Disturb (*n* = 8)	Pseudb. Palsy (*n* = 5)	Demen (*n* = 18)	Gait Distur (*n* = 4)	FH ^a^ (*n* = 25)	LETP ^b^ (*n* = 11)	GOM (*n* = 10)	Author
1	p.R61W	USA	-	Yes	No	No	Yes	20	Yes	Yes	-	-	-	-	-	Yes	No	*	Brass
2	p.R75P	Korea	M	-	Yes	No	No	53	-	Yes	-	-	-	Yes	-	Yes	-	Yes	Kim
3	p.R75P	Korea	F	No	No	No	No	47	-	Yes	-	-	-	-	-	Yes	-	-	Kim
4	p.R75P	Korea	M	No	Yes	No	No	65	-	Yes	-	-	-	Yes	-	Yes	-	-	Kim
5	p.R75P	Japan	F	-	-	-	-	-	-	Yes	Yes	Yes	Yes	-	-	Yes	No	-	Mizuno
6	p.R75P	Japan	F	-	-	-	-	-	-	Yes	Yes	-	Yes	-	-	Yes	No	Yes	Mizuno
7	p.R75P	China	M	-	-	-	-	34	-	Yes	-	Yes	-	-	-	No	No	Yes	Wang
8	p.D80G	Germany	F	No	No	No	No	-	No	Yes	-	Yes	-	Yes	Yes	Yes	Yes	*	Wollenweber
9	p.R107W	Germany	-	-	-	-	-	-	Yes	-	-	-	-	Yes	-	Yes	-	-	Ungaro
10	p.G149V	China	F	No	No	No	No	39	-	Yes	-	-	-	-	-	Yes	No	-	Ge
11	p.Q151E	Italy	-	-	-	-	-	-	Yes	-	-	-	-	Yes	-	Yes	-	-	Ungaro
12	p.Q151E	Spain	-	-	-	-	-	-	-	Yes	-	-	-	-	-	-	-	-	Ampuero
13	p.H170R	Spain	-	-	-	-	-	-	-	Yes	-	-	-	-	-	-	-	-	Ampuero
14	p.H170R	Oceania	-	-	-	-	-	-	-	-	-	-	-	-	-	-	-	-	Roy
15	p.A198T	Italy	-	-	-	-	-	-	Yes	-	-	-	-	Yes	-	Yes	-	-	Ungaro
16	p.A202V	Oceania	F	-	-	-	-	-	-	-	-	-	-	-	-	-	-	-	Roy
17	p.R207H	Italy	-	-	-	-	-	-	Yes	-	-	-	-	Yes	-	Yes	-	-	Ungaro
18	p.R213K	Japan	M	-	No	No	No	63	Yes	Yes	No	-	Yes	Yes	Yes	Yes	-	-	Uchino
19	p.R213K	Japan	M	-	No	No	No	10	Yes	Yes	-	Yes	Yes	Yes	Yes	Yes	-	Yes	Santa
20	p.V237M	Japan	F	-	No	No	No	71	-	Yes	No	-	No	Yes	Yes	Yes	-	-	Uchino
21	p.V252M	Russia	-	-	No	No	-	-	-	-	-	-	-	-	-	-	-	-	Abramycheva
22	p.E309K	Italy	-	-	-	-	-	-	Yes	-	-	-	-	Yes	-	Yes	-	-	Ungaro
23	p.S497L	Russia	-	-	No	No	-	-	-	-	-	-	-	-	-	-	-	-	Abramycheva
24	p.T577A	Portugal	-	-	-	-	-	-	-	-	-	-	-	-	-	-	-	-	Ferreira
25	p.R592S	Italy	-	-	-	-	-	-	Yes	-	-	-	-	Yes	-	Yes	-	-	Ungaro
26	p.V644D	Italy	-	-	-	-	-	-	Yes	-	-	-	-	Yes	-	Yes	-	-	Ungaro
27	p.S978R	Portugal	F	-	-	-	-	-	-	Yes	Yes	Yes	-	Yes	-	-	-	-	Ferreira
28	p.A1020P	Germany	F	-	Yes	-	-	Adolesc	Yes	-	-	-	-	Yes	-	Yes	No	Yes	Scheid
29	p.A1020P	Germany	F	-	Yes	-	-	-	-	-	-	Yes	-	-	-	Yes	No	No	Scheid
30	p.T1098S	China	M	-	-	-	-	39	-	Yes	-	Yes	-	Yes	-	Yes	No	Yes	Wang
31	p.H1133Q	Russia	-	-	No	No	-	-	-	-	-	-	-	-	-	-	-	-	Abramycheva
32	p.H1235L	Italy	-	-	-	-	-	-	Yes	-	-	-	-	Yes	-	Yes	-	-	Ungaro
33	p.L1515P	France	F	No	No	No	No	35	Yes	Yes	-	-	-	-	-	Yes	No	No	Fouillade
34	p.V1762M	Italy	F	Yes	-	-	Yes	Childhd	Yes	-	-	Yes	-	No	-	Yes	No	No	Bersano
Perc	-	-	-	29%	27%	0%	18%	-	93%	100%	60%	100%	80%	94%	100%	96%	9%	40%	-

Numb: Number; Smoker: current/past smoker; HT: hypertension; DM: diabetes mellitus; Dyslip: dyslipidaemia; Clin. Onset: Clinical Onset; Psych.distur: psychiatric disturbance; Pseudb.palsy: pseudobulbar palsy; Dem.: dementia; Gait distur: gait disturbance; GOM: Granular osmiophilic material; M: male; F: female; Adolesc: adolescence; Childhd: childhood; Perc: percentage; ^a^ FH: family history; ^b^ LE TP: leukoencephalopathy with anterior temporal pole involvement; * GOM found in proband’s siblings, who were symptomatic.

**Table 3:** Summary of the main characteristics of cysteine-sparing *NOTCH3* missense mutations.

Number	Mutation	Typical Clinical CADASIL Syndrome	WMH	Whole Exon Analysis	Mutation	GOM	Author
1	p.R61W	Yes	Yes	Yes	Yes	*	Brass
2	p.R75P	Yes	Yes	No	Yes	Yes	Kim
3	p.R75P	Yes	Yes	No	Yes	-	Kim
4	p.R75P	Yes	Yes	No	Yes	-	Kim
5	p.R75P	Yes	Yes	Yes	Yes	-	Mizuno
6	p.R75P	Yes	Yes	Yes	Yes	Yes	Mizuno
7	p.R75P	Yes	Yes	No	Yes	Yes	Wang
8	p.D80G	Yes	Yes	Yes	Yes	*	Wollenweber
9	p.R107W	Yes	Yes	No	Yes	-	Ungaro
10	p.G149V	Yes	Yes	Yes	Yes	-	Ge
11	p.Q151E	Yes	Yes	No	Yes	-	Ungaro
12	p.Q151E	Yes	Yes	No	Yes	-	Ampuero
13	p.H170R	Yes	Yes	No	No	-	Ampuero
14	p.H170R	Yes	NS	No	No	-	Roy
15	p.A198T	Yes	Yes	No	Yes	-	Ungaro
16	p.A202V	Yes	NS	No	Yes	-	Roy
17	p.R207H	Yes	Yes	No	Yes	-	Ungaro
18	p.R213K	Yes	Yes	NS	Yes	-	Uchino
19	p.R213K	Yes	Yes	Yes	Yes	Yes	Santa
20	p.V237M	Yes	Yes	NS	Yes	-	Uchino
21	p.V252M	Yes	Yes	No	Yes	-	Abramycheva
22	p.E309K	Yes	Yes	No	Yes	-	Ungaro
23	p.S497L	Yes	NS	No	No	-	Abramycheva
24	p.T577A	NS	NS	NS	Yes	-	Ferreira
25	p.R592S	Yes	Yes	No	Yes	-	Ungaro
26	p.V644D	Yes	Yes	No	Yes	-	Ungaro
27	p.S978R	Yes	Yes	NS	Yes	-	Ferreira
28	p.A1020P	Yes	Yes	NS	No	Yes	Scheid
29	p.A1020P	Yes	Yes	NS	No	No	Scheid
30	p.T1098S	Yes	Yes	No	Yes	Yes	Wang
31	p.H1133Q	Yes	NS	No	No	-	Abramycheva
32	p.H1235L	Yes	Yes	No	No	-	Ungaro
33	p.L1515P	Yes	Yes	Yes	Yes	No	Fouillade
34	p.V1762M	Yes	Yes	Yes	Yes	No	Bersano

WMH: white matter hyperintensities; GOM: Granular osmiophilic material; NS: not specified; * GOM were found in proband’s sibling, who was symptomatic.

**Table 4:** Polyphen-2 results and domain localization of the selected mutations.

Mutation	Score	Confidence	Domain	Prediction
R61W	0.773	Sensitivity: 0.76	EGF-like 1	Possibly damaging
		Especificity: 0.86		
R75P	0.884	Sensitivity: 0.71	EGF-like 1	Possibly damaging
		Especificity: 0.89		
D80G	0.694	Sensitivity: 0.78	EGF-like 2	Possibly damaging
		Especificity: 0.85		
R213K	0.171	Sensitivity: 0.89	EGF-like 5	Benign
		Especificity: 0.72		

## Abbreviations

CADASIL	Cerebral autosomal dominant arteriopathy with subcortical infarcts and Leukoencephalopathy
ECD	Extracellular domain
EGF	Epidermal growth factor
ICD	Intracellular domain
MRI	Magnetic resonance imaging
WMH	White matter hyperintensities
GOM	Granular osmiophilic material
MAF	Minor allele frequency
ExAC	Exome aggregation consortium
